# Mapping the allosteric network within a SH3 domain

**DOI:** 10.1038/s41598-019-44656-8

**Published:** 2019-06-04

**Authors:** Francesca Malagrinò, Francesca Troilo, Daniela Bonetti, Angelo Toto, Stefano Gianni

**Affiliations:** Istituto Pasteur - Fondazione Cenci Bolognetti, Dipartimento di Scienze Biochimiche “A. Rossi Fanelli” and Istituto di Biologia e Patologia Molecolari del CNR, Sapienza Università di Roma, 00185 Rome, Italy

**Keywords:** Proteins, Kinetics

## Abstract

SH3 domains are very abundant protein-protein interactions modules, involved in the regulation of several cellular processes. Whilst they have been associated to allosteric communication pathways between contiguous domains in multi-domain proteins, there is lack of information regarding the intra-domain allosteric cross-talk within the SH3 moiety. Here we scrutinize the presence of an allosteric network in the C-terminal SH3 domain of Grb2 protein, upon binding the Grb2-associated binding 2 protein. To explore allostery, we performed double mutant cycle analysis, a powerful quantitative approach based on mutagenesis in conjunction with kinetic experiments. Data reveal the presence of an unexpected allosteric sparse network that modulates the affinity between the SH3 domain and its physiological partner.

## Introduction

Src homology 3 (SH3) domains are among the most abundant protein-protein interaction (PPI) modules, found in a large amount of human proteins and implicated in the control of critical cellular processes, such as cell scaffolding, signal transduction, cytoskeletal modification and proliferation^1–4^. From a structural point of view, the SH3 domains comprise of approximately 60 amino acid residues and display a characteristic β-barrel fold formed by five or six β-strands arranged as two antiparallel β-sheets^5,6^. Generally, these adapter domains recognize substrates with proline-rich motifs typically organized in a polyproline type II (PPII) helix; the interactions are highly specific and promote the assemblage of the molecular complexes involved in signaling transduction^2^. Furthermore, SH3 domains are also able to regulate the catalytic activity or the binding site accessibility of the proteins containing them.

Despite SH3 domains share a highly conserved structure and a relatively simple binding pocket, they are nevertheless very specific^7–9^. Among the numerous potential proline-rich binding targets, the distinct role of each SH3 domain in the crowded cellular environment demands precise interactions, while avoiding potentially harmful non-specific reactions. One possible strategy to achieve such specificity is to modulate binding affinity through allosteric effects, whereby residues far from the binding pocket may tune the energetics of binding to a specific ligand.

Whilst allosteric effects in SH3 domains have been previously explored to investigate the structural communication between contiguous domains in complex multidomain proteins^10–14^, there is lack of information regarding the intra-domain allosteric cross-talk within the SH3 moiety. One of the most powerful methods to describe intra-domain allostery is the measurement of inter-residue energetic communication upon binding through the so-called double mutants cycle analysis. This experimental approach provides a quantitative measurement of the energetic coupling between distal residues which are involved in the binding reaction.

By employing double mutant cycles^15^, we provide here the first demonstration of an allosteric network within an SH3 domain. In particular, we investigated the energetic coupling upon binding between the C-terminal SH3 domain of the adapter protein Grb2 and the intrinsically disordered protein Gab2^16,17^. The experimental results allowed us to map the presence of an allosteric network that might be implicated in balancing the selectivity of this domain for its physiological partners.

## Results and Discussion

### Double mutant cycles as a tool to investigate protein allostery

The classical view of protein allostery relies on a detectable conformational change, regulating the functions of the active site(s)^18,19^. However, allosteric modulation of macromolecule might also be more elusive and allostery might be at play even in the absence of a prominent conformational modification, involving either dynamic^20,21^, chronosteric effects^22,23^ or long range interactions modulating amyloidogenic effects^24^. In these cases, the characterization of the allosteric contribution in a binding reaction demands a careful experimental investigation, as it might be elusive to the structural characterization of the protein.

A powerful method to investigate protein allostery is represented by the measurement of the energetic coupling between the ligand and residues of the protein that are spatially distant from the binding site^15,25–27^. In fact, rather than observing detectable conformational changes, it is possible to define and quantify interaction networks that modulate the allosteric communication within the protein moiety. The quantification of these allosteric connections allows to pinpoint the structural elements critical for the binding processes and, therefore, to obtain a description in energetic terms of such structural communication.

Double mutant cycle analysis is a mighty method based on mutagenesis and measurements of binding free energies, providing a quantitative description of the allosteric energetic connections upon binding^15^. The experimental approach can be described by considering a system *P-AB* containing two residues under investigation *A* and *B*. The measurement of the energetic contributions of these residues to a given reaction might be explored by producing the deletion mutants *P-A*, where *B* is deleted, *P-B*, where *A* is deleted, and *P*, where both residues are deleted. Quantitatively, the changes in free energy for single mutants at positions *A* and *B* can be then calculated assuming the following equations:

1 $${\rm{\Delta }}{\rm{\Delta }}{{G}}_{{\rm{P}}-{\rm{AB}}}{\to }_{{\rm{P}}-{\rm{B}}}={\rm{\Delta }}{{G}}_{{\rm{P}}-{\rm{AB}}}-{\rm{\Delta }}{{G}}_{{\rm{P}}-{\rm{B}}}$$

2 $$\Delta {\rm{\Delta }}{{G}}_{{\rm{P}}-{\rm{AB}}}{\to }_{{\rm{P}}-{\rm{A}}}={\rm{\Delta }}{{G}}_{{\rm{P}}-{\rm{AB}}}-{\rm{\Delta }}{{G}}_{{\rm{P}}-{\rm{A}}}$$

3 $${\rm{\Delta }}{\rm{\Delta }}{{G}}_{{\rm{P}}-{\rm{AB}}}{\to }_{{\rm{P}}}={\rm{\Delta }}{{G}}_{{\rm{P}}-{\rm{AB}}}-{\rm{\Delta }}{{G}}_{{\rm{P}}}$$

By applying a squared thermodynamic cycle, it may be noted that the change in energy upon deleting both A and B (ΔΔ*G*_P-__AB_→_P_) should equal the sum of the change in energies for the single mutants (ΔΔ*G*_P-AB_→_P-B_ + ΔΔ*G*_P-AB_→_P-A_), if *A* and *B* do not interact energetically. The coupling energy ΔΔΔ*G* can be derived subtracting the effects of the single mutants to that of the corresponding double mutant:

4 $${\rm{\Delta }}{\rm{\Delta }}{\rm{\Delta }}G={\rm{\Delta }}{\rm{\Delta }}{G}_{{\rm{P}}-{\rm{AB}}}{\to }_{{\rm{P}}}-{\rm{\Delta }}{\rm{\Delta }}{G}_{{\rm{P}}-{\rm{AB}}}{\to }_{{\rm{P}}-{\rm{B}}}-{\rm{\Delta }}{\rm{\Delta }}{G}_{{\rm{P}}-{\rm{AB}}}{\to }_{{\rm{P}}-{\rm{A}}}$$

The ΔΔΔ*G* measures the energetic strength of the interaction between positions A and B and when its value is different from zero represents the signature for the two residues to interact energetically.

In the case of protein-protein interaction, double mutant cycles can be promptly applied to measure the functional role of residues that are not physically located in the binding site of a protein. In fact, by considering the example described above, if the residue A is located in one of the interacting protein and B in the other, the energetic coupling between A and B can be associated to the strength of their interaction, independently on whether they physically interact in the complex^25,27^. For these reasons, this methodology represents an ideal technique to monitor protein allostery quantitatively.

### Experimental set up to investigate allostery on SH3 by double mutant cycles

In order to investigate the allosteric network in a SH3 domain we resorted to perform a double mutant cycle analysis of the C-terminal SH3 domain of Grb2 and a peptide mimicking its physiological ligand Gab2, denoted as Gab2_503–524_^16,17^. In analogy to a typical ligand for an SH3 domain^3^, Gab2_503–524_ corresponds to a proline rich sequences that undergoes a disorder to order transition upon binding, forming a polyproline type II helix. As briefly recalled above, the double mutant cycle methodology relies on the measurement of changes in binding free energy upon mutation (ΔΔ*G*). When perturbing positions that are directly involved in binding, it is therefore of critical importance to design the mutants carefully and focus on those positions that display a ΔΔ*G* that is high enough to be experimentally determined but low enough not to perturb binding. In fact, if a reference mutation destabilizes the complex too much, the propagation of the associated errors may jeopardize the quantitative analysis of the corresponding ΔΔΔ*G*, see for example ref.^28^. In the case of Gab2_503–524_, it was recently shown that mutations P510A and P512A display a detectable effect in the stabilization of the complex, whilst the other amino acids involved in binding appear to display a dramatic effect (with mutations of P511A, R515A, K518A and P519A yielding to a very pronounced change in K_D_)^16,17^. In an effort to detect the presence of allosteric networks in an SH3 domain, we therefore subjected the C-terminal SH3 domain of Grb2 to extensive site directed mutagenesis and challenged 18 variants of with (i) WT Gab2_503–524_, (ii) P510A Gab2_503–524_ and (iii) P512A Gab2_503–524_. In analogy to what previously discussed in protein folding studies, the mutants were designed following standard procedures, which has been extensively discussed previously by Fersht and co-workers^29^. In turn, each mutant was constructed to introduce a small deletion of the side-chain without changing the steric properties of the residues. Importantly, previous detailed investigation on the folding and stability of these variants of SH3 revealed that they all well-folded stable proteins displaying a similar (un)folding co-operativity compared to wild type SH3^30^, confirming that none of the mutations had a relevant effect on the structure of the domain.

**Figure 1:**
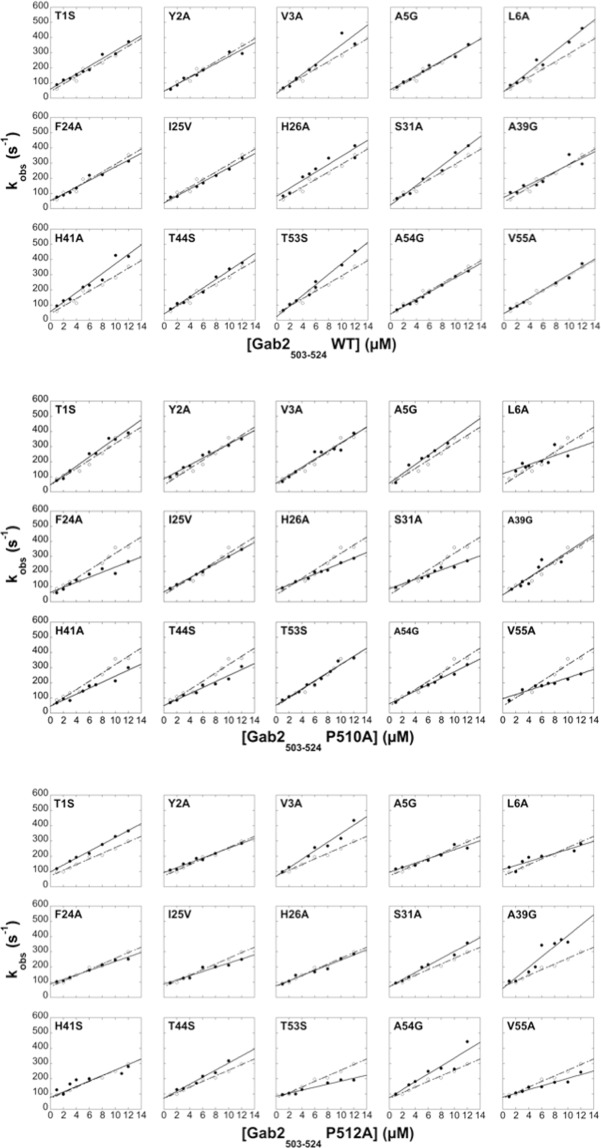
Kinetics of binding of C-SH3 Grb2 wild-type (dashed line, empty circles) and its site-directed mutants (black line, full circles) with Gab2_503–524_ wild-type (top), Gab2_503–524_ P510A (centre) and Gab2_503–524_ P512A (bottom). The experiments were carried out in pseudo-first order condition in 50 mM HEPES buffer, 0.5 M NaCl, pH 7.0, at 10 °C. The linear behaviour of the plot of the observed rate constant (*k*_obs_) versus Gab2_503–524_ concentration is consistent with a two-state nature of the reaction.

We carried out pseudo-first-order binding experiments at 10 °C using a stopped-flow apparatus and mixing a constant concentration of C-SH3 Grb2 (0.5 μM) with increasing concentrations of Gab2_503–524_ (2–12 μM). As described previously, the binding reaction was monitored following changes in fluorescence upon binding of Trp35 and Trp36 residues of C-SH3 Grb2. The resulting kinetic trace was fitted to a single exponential function to obtain the observed rate constant *k*_obs_. Under all the investigated conditions, observed kinetics were consistent with a single-exponential behavior, suggesting the lack of accumulation of intermediates. Figure 1 compares the kinetic data obtained for the binding of wild type C-SH3 Grb2 and its site-directed mutants to WT Gab2_503–524_, P510A Gab2_503–524_ and P512A Gab2_503–524_ respectively. It is evident that, in all cases, a plot of the observed rate constant (*k*_obs_) as function of Gab2_503–524_ concentration is consistent with a linear behaviour, further supporting a two-state mechanism at the explored experimental condition. By applying the pseudo-first order approximation, the association and dissociation rate constants were obtained from the dependence of the observed rate constant on [Gab2_503–524_].

Interestingly, several mutations displayed a detectable change in K_D_, despite the position being distant from the binding pocket (Table 1). For example, mutation T53S showed an increase in about 10 fold in K_D_ when challenged with P512A Gab2_503–524_, despite not being in direct contact with the peptide. This finding suggests the presence of an allosteric behaviour in C-SH3 Grb2, which cannot be rationalized in a simple manner from a structural point of view and demands careful investigation by taking advantange of coupling energies, as detailed below.

**Table 1:** Kinetic parameters and coupling free energies (ΔΔΔG) of the binding reaction of C-SH3 Grb2 wild-type and its site-directed mutants with Gab2_503–524_ wild-type, Gab2_503–524_ P510A and Gab2_503–524_ P512A.

C-SH3 Grb2	Gab2_503–524_ WT			Gab2_503–524_ P510A					Gab2_503–524_ P512A				
mutant	*k*_on_ (µM^−1^ s^−1^)	*k*_off_ (s^−1^)	*K*_D_ (µM)	*k*_on_ (µM^−1^ s^−1^)	*k*_off_ (s^−1^)	*K*_D_ (µM)	∆∆∆G (kcal mol^−1^)	Distance (Å)	*k*_on_ (µM^−1^ s^−1^)	*k*_off_ (s^−1^)	*K*_D_ (µM)	∆∆∆G (kcal mol^−1^)	Distance (Å)
WT	25.0 ± 1.8	40 ± 4	1.7 ± 0.1	27.4 ± 2.2	50 ± 4	1.6 ± 0.1	—	21.4	18.5 ± 1.0	70 ± 7	3.8 ± 0.1	—	23.8
T1S	25.3 ± 1.6	60 ± 6	2.3 ± 0.1	30.6 ± 1.9	50 ± 5	1.5 ± 0.1	0.22 ± 0.08	23.0	22.5 ± 0.8	100 ± 6	4.3 ± 0.1	0.11 ± 0.06	27.4
Y2A	22.9 ± 2.1	50 ± 5	2.0 ± 0.1	22.9 ± 1.3	90 ± 10	3.7 ± 0.1	−0.37 ± 0.07	15.7	16.0 ± 1.0	90 ± 6	5.8 ± 0.1	−0.13 ± 0.07	18.6
V3A	32.2 ± 3.9	30 ± 3	0.9 ± 0.2	26.6 ± 2.9	60 ± 6	2.1 ± 0.2	−0.48 ± 0.12	10.4	27.9 ± 2.5	70 ± 7	2.4 ± 0.1	−0.07 ± 0.11	16.9
A5G	24.0 ± 1.4	60 ± 10	2.3 ± 0.2	30.5 ± 3.4	60 ± 6	1.9 ± 0.2	0.09 ± 0.09	9.9	14.7 ± 2.0	100 ± 14	6.4 ± 0.2	−0.13 ± 0.07	13.1
L6A	33.9 ± 2.4	40 ± 20	1.3 ± 0.4	14.9 ± 5.0	120 ± 30	8.1 ± 0.4	−1.06 ± 0.18	5.1	13.1 ± 2.3	110 ± 16	8.5 ± 0.2	−0.62 ± 0.18	4.9
F7A	—	—	—	—	—	—	—	10.4	—	—	—	—	10.2
F19A	—	—	—	—	—	—	—	18.6	—	—	—	—	24.6
F24A	22.3 ± 1.8	50 ± 5	2.3 ± 0.1	16.7 ± 2.3	60 ± 6	3.7 ± 0.2	−0.28 ± 0.07	13.7	14.7 ± 0.9	90 ± 6	6.0 ± 0.1	−0.07 ± 0.06	18.0
I25V	23.3 ± 1.3	40 ± 10	1.6 ± 0.3	23.4 ± 0.5	60 ± 3	2.7 ± 0.1	−0.32 ± 0.11	25.3	13.7 ± 1.6	90 ± 9	6.4 ± 0.2	−0.32 ± 0.11	28.0
H26A	26.3 ± 3.5	80 ± 8	3.1 ± 0.2	17.7 ± 0.9	80 ± 7	4.3 ± 0.1	−0.21 ± 0.07	25.7	16.9 ± 1.5	80 ± 10	4.4 ± 0.2	0.26 ± 0.07	23.1
S31A	32.5 ± 2.0	20 ± 2	0.7 ± 0.1	15.5 ± 1.2	90 ± 8	5.6 ± 0.1	−1.20 ± 0.11	21.7	23.1 ± 1.3	70 ± 9	3.0 ± 0.1	−0.38 ± 0.11	24.9
A39G	21.5 ± 3.9	70 ± 7	3.4 ± 0.2	28.5 ± 6.4	50 ± 4	1.6 ± 0.2	0.42 ± 0.11	16.4	34.6 ± 4.9	60 ± 6	1.7 ± 0.2	0.86 ± 0.09	23.0
H41A	31.6 ± 3.0	60 ± 5	1.8 ± 0.1	19.8 ± 1.8	50 ± 5	2.3 ± 0.1	−0.17 ± 0.08	23.0	18.0 ± 2.9	80 ± 8	4.3 ± 0.2	−0.04 ± 0.07	24.6
T44S	28.6 ± 1.5	40 ± 5	1.4 ± 0.1	19.8 ± 1.7	50 ± 5	2.5 ± 0.1	−0.32 ± 0.08	4.6	23.2 ± 2.3	70 ± 15	3.0 ± 0.2	0.04 ± 0.09	5.2
Y51A	—	—	—	—	—	—	—	14.2	—	—	—	—	18.0
T53S	34.9 ± 1.8	20 ± 2	0.7 ± 0.1	26.9 ± 1.7	50 ± 5	1.9 ± 0.1	−0.62 ± 0.12	17.7	9.8 ± 1.0	90 ± 7	8.6 ± 0.1	−0.99 ± 0.11	20.7
A54G	24.0 ± 0.7	40 ± 5	1.7 ± 0.1	21.1 ± 1.1	60 ± 8	2.9 ± 0.1	−0.33 ± 0.08	17.6	25.9 ± 4.4	80 ± 8	2.9 ± 0.2	0.15 ± 0.08	21.9
V55A	25.7 ± 1.6	40 ± 4	1.7 ± 0.1	13.8 ± 1.6	100 ± 10	6.9 ± 0.2	−0.81 ± 0.07	21.4	12.3 ± 1.4	80 ± 9	6.4 ± 0.2	−0.29 ± 0.07	23.8

### Mapping allosteric communication on the structure of the SH3 domain

The coupling free energies (ΔΔΔG) of each mutated residue of the C-SH3 domain with both prolines (Pro510 and Pro512) of Gab2_503–524_ were then quantified using the experimental association (*k*_on_) and dissociation (*k*_off_) rate constants obtained by kinetics (Table 1). Interestingly, we found that six residues (Val3, Leu6, Ser31, Ala39, Thr53 and Val55) showed a detectable ΔΔΔG upon mutation and binding with Gab2 P510A, i.e. with a value of ΔΔΔG > 0.4 kcal mol^−1^. More to the point, four of these residues, Leu6, Ser31, Ala39 and Thr53, were also found energetically coupled with the Pro512, with a ΔΔΔG of 0.6 ± 0.2, 0.4 ± 0.1, 0.9 ± 0.1 and 1.0 ± 0.1 kcal mol^−1^ respectively. Upon mutation of residues Phe7, Phe19 and Tyr51 the binding reaction does not take place, suggesting that these residues play a key role of these amino acids in such reaction.

**Figure 2:**
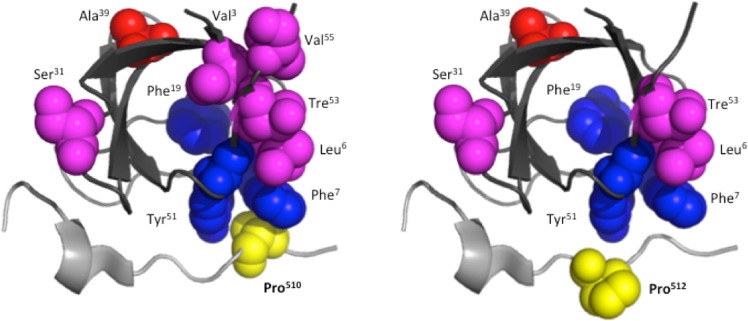
Cartoon representation of the complex between the C-SH3 Grb2 and Gab2_503–524_ (drawn in Pymol; PDB code: 2VWF). C-SH3 Grb2 domain is represented in black, Gab2_503–524_ in gray while mutated residues are represented as spheres on the structure of the complex. The energetic coupling between the prolines of Gab2 in pos 510 (left; yellow sphere) and 512 (right; yellow sphere) with the residues of C-SH3 Grb2 is highlighted as magenta spheres in case of negative values of ΔΔΔG and as red spheres in case of positive values of ΔΔΔG. Blue spheres highlight mutations that abrogate binding.

It is of interest to study the structural distribution of the residues displaying a detectable ΔΔΔG or a pronounced effect on binding (Fig. 2). In fact, whilst Phe9 and Tyr51 are physically located in the binding pocket, Val3, Leu6, Ser31, Ala39, Thr53 and Val55 do not engage any contact with Gab2_503–524_. Because none of these residue is directly located in the binding pocket of C-SH3, it may be concluded that they play an allosteric role in the recognition of Gab2_503–524_ and represent a sparse network within the SH3 domain that regulates binding. This finding parallels earlier work on PDZ domains^27^, where the presence of a sparse network could not be rationalized easily on the structure of the domains. Therefore, allosteric sparse network might represent a general feature in protein-protein recognition domain and demand additional work.

### Allostery and selectivity – on the significance of positive and negative ΔΔΔG values

One of the plausible mechanisms whereby protein domain selectivity is achieved is to account for long range allosteric networks^7^. In fact, when and if an adaptor domain family displays a highly conserved structure and a relatively simple binding pocket, the fine-tuning of its selectivity may be achieved by long range interactions that are modulated by residues that are physically distant from the binding pocket. As previously shown on PDZ domains^27^, a characteristic signature of such scenario is represented by the positive sign in the ΔΔΔG values. In other words, if a mutation of core residue in the protein will affect the binding of the peptide such that the effect of a perturbation in the peptide will be smaller in the mutant as compared to the wild type protein, it follows that the sequence of the wild type is optimized to bind the wild type sequence of the peptide. On the contrary of what previously observed for PDZ domains^27^, the calculated values reported in Table 1 do not show a clear prevalence of positive ΔΔΔG values. We speculate that this arises from the complex role of the C-SH3 domain, which is involved in several cellular pathways and has been suggested to interact with different ligands^31–33^.

## Conclusions

The allosteric regulation of protein is a general mechanism whereby physically distant residues regulate the functionality of the active site through conformational changes, dynamic or chronosteric effects. In the case of SH3 domains, whilst they have been often associated to allosteric pathways, the intra-domain communication within residues has been poorly explored to date. The employment of extensive site-directed mutagenesis and double mutants cycles allowed us to map an allosteric sparse network that regulates the recognition between an SH3 domain and a peptide mimicking its physiological partner. Future work on other SH3 domains will further clarify the importance and generality of this network.

## Materials and Methods

C-SH3 Grb2 wild-type and its site-directed mutants were produced as previously reported^30^. Kinetic experiments of binding were performed on a single-mixing SX-18 stopped-flow instrument (Applied Photophysics), recording the change of fluorescence emission. The excitation wavelength used was 280 nm while the fluorescence emission was collected using a 320-nm cut-off glass filter. The binding experiments were carried out at 10 °C in pseudo-first order condition mixing a constant concentration of C-SH3 Grb2 in the wild-type and mutated forms (0.5 μM) versus increasing concentrations of Gab2_503–524_ wild-type and its mutants P510A and P512A (ranging from 1 to 12 µM). For all measurements the buffer used was 50 mM HEPES, 0.5 M NaCl, pH 7.0. The observed rate constants (*k*_obs_) were calculated from the average of 3–6 single traces and by fitting of the time-course for binding using a single exponential equation.

## References

[1] Kurochkina N, Guha U (2013). SH3 domains: modules of protein-protein interactions. Biophys. Rev..

[2] Saksela K, Permi P (2012). SH3 domain ligand binding: What’s the consensus and where’s the specificity?. FEBS Lett..

[3] Kay B (2012). SH3 domains come of age. FEBS Lett..

[4] Teyra J, Sidhu SS, Kim PM (2012). Elucidation of the binding preferences of peptide recognition modules: SH3 and PDZ domains. FEBS Lett..

[5] Yu H (1992). Solution structure of the SH3 domain of Src and identification of its ligand-binding site. Science.

[6] Musacchio A, Noble M, Pauptit R, Wierenga R, Saraste M (1992). Crystal structure of a Src-homology 3 (SH3) domain. Nature.

[7] Fernandez-Ballester G, Blanes-Mira C, Serrano L (2004). The tryptophan switch: changing ligand-binding specificity from type I to type II in SH3 domains. J. Mol. Biol..

[8] Cesareni G, Panni S, Nardelli G, Castagnoli L (2002). Can we infer peptide recognition specificity mediated by SH3 domains?. FEBS Lett..

[9] Panni, S., Dente, L. & Cesareni, G. *In vitro* evolution of recognition specificity mediated by SH3 domains reveals target recognition rules. *J*. *Biol*. *Chem*. **277** (2002). 10.1074/jbc.M109788200 11929862

[10] Shah NH, Amacher JF, Nocka LM, Kuriyan J (2018). The Src module: an ancient scaffold in the evolution of cytoplasmic tyrosine kinases. Crit. Rev. Biochem. Mol. Biol..

[11] Yang F (2018). Allosteric mechanisms underlie GPCR signaling to SH3-domain proteins through arrestin. Nat. Chem. Biol..

[12] Dionne, U. *et al*. Direct Phosphorylation of SRC Homology 3 Domains by Tyrosine Kinase Receptors Disassembles Ligand-Induced Signaling Networks. *Mol*. *Cel*. **70** (2018). 10.1016/j.molcel.2018.05.013 PMC6014926 29910111

[13] Register AC, Chakraborty S, Maly DJ (2017). Allosteric Modulation of Src Family Kinases with ATP-Competitive Inhibitors. Methods Mol. Biol..

[14] Marcette J, Hood IV, Johnston CA, Doe CQ, Prehoda KE (2009). Allosteric control of regulated scaffolding in membrane-associated guanylate kinases. Biochemistry.

[15] Horovitz A (1996). Double-mutant cycles: a powerful tool for analyzing protein structure and function. Fold. Des..

[16] Krieger JM (2014). Conformational recognition of an intrinsically disordered protein. Biophys. J..

[17] Toto A, Bonetti D, De Simone A, Gianni S (2017). Understanding the mechanism of binding between Gab2 and the C terminal SH3 domain from Grb2. Oncotarget.

[18] Monod J, Changeux J-P, Jacob F (1963). Allosteric proteins and cellular control systems. J. Mol. Biol..

[19] Monod J, Wyman J, Changeux J-P (1965). On the nature of allosteric transitions: A plausible model. J. Mol. Biol..

[20] Cooper A, Dryden DT (1984). Allostery without conformational change. A plausible model. Eur. Biophys. J..

[21] Nussinov R, Tsai CJ (2015). Allostery without a conformational change? Revisiting the paradigm. Curr. Opin. Struct. Biol..

[22] Hilser VJ, Anderson JA, Motlagh HN (2015). Allostery vs. “allokairy”. Proc. Natl. Acad. Sci. USA.

[23] Ascenzi P, Gianni S (2013). Functional role of transient conformations: Rediscovering “chronosteric effects” thirty years later. IUBMB Life.

[24] Le Marchand T (2018). Conformational dynamics in crystals reveal the molecular bases for D76N beta-2 microglobulin aggregation propensity. Nat. Commun..

[25] Serrano L, Horovitz A, Avron B, Bycroft M, Fersht AR (1990). Estimating the contribution of engineered surface electrostatic interactions to protein stability by using double-mutant cycles. Biochemistry.

[26] Horovitz A, Fersht AR (1990). Strategy for analysing the co-operativity of intramolecular interactions in peptides and proteins. J. Mol. Biol..

[27] Gianni S (2011). Sequence-specific long range networks in PSD-95/discs large/ZO-1 (PDZ) domains tune their binding selectivity. J. Biol. Chem..

[28] Chi CN (2008). Reassessing a sparse energetic network within a single protein domain. Proc. Natl. Acad. Sci. USA.

[29] Fersht AR, Sato S (2004). Phi-value analysis and the nature of protein-folding transition states. Proc. Natl. Acad. Sci. USA.

[30] Troilo, F. *et al*. Folding Mechanism of the SH3 Domain from Grb2. *J*. *Phys*. *Chem*. *B*, Aug 23. 10.1021/acs.jpcb.1028b06320. [Epub ahead of print], (2018). 10.1021/acs.jpcb.8b06320 30091591

[31] McDonald CB (2013). Allostery mediates ligand binding to Grb2 adaptor in a mutually exclusive manner. J. Mol. Recognit..

[32] Gril B (2007). Grb2-SH3 ligand inhibits the growth of HER2+ cancer cells and has antitumor effects in human cancer xenografts alone and in combination with docetaxel. Int. J. Cancer.

[33] Weinger JG (2008). In brain, Axl recruits Grb2 and the p85 regulatory subunit of PI3 kinase; *in vitro* mutagenesis defines the requisite binding sites for downstream Akt activation. J. Neurochem..

